# Inhibition of Macropinocytosis Enhances the Sensitivity of Osteosarcoma Cells to Benzethonium Chloride

**DOI:** 10.3390/cancers15030961

**Published:** 2023-02-02

**Authors:** Haichao Xia, Yanran Huang, Lulu Zhang, Lijuan Luo, Xiaoxuan Wang, Qiuping Lu, Jingtao Xu, Chunmei Yang, Habu Jiwa, Shiqiong Liang, Liping Xie, Xiaoji Luo, Jinyong Luo

**Affiliations:** 1 Key Laboratory of Clinical Laboratory Diagnostics, Ministry of Education, Chongqing Medical University, Chongqing 400016, China; 2 Department of Orthopedics, The First Affiliated Hospital of Chongqing Medical University, Chongqing 400016, China

**Keywords:** osteosarcoma, benzethonium chloride, macropinocytosis, ERK1/2 signaling pathway

## Abstract

**Simple Summary:**

Osteosarcoma (OS) is a primary malignant tumor of bone. Macropinocytosis, a form of non-selective nutrient endocytosis, has received increasing attention as a novel target for cancer therapy, yet its role in OS cells remains obscure. Benzethonium chloride (BZN) is an FDA-approved antiseptic and bactericide with broad-spectrum anticancer effects. Here, we described that BZN suppressed the proliferation, migration, and invasion of OS cells in vitro and in vivo. Mechanistically, BZN repressed the ERK1/2 signaling pathway, and the ERK1/2 activator partially neutralized the inhibitory effect of BZN on OS cells. Subsequently, we demonstrated that OS cells might employ macropinocytosis as a compensatory survival mechanism in response to BZN. Remarkably, macropinocytosis inhibitors enhanced the anti-OS effect of BZN in vitro and in vivo. In conclusion, our results suggest that BZN may inhibit OS cells by repressing the ERK1/2 signaling pathway and propose a potential strategy to enhance the BZN-induced inhibitory effect by suppressing macropinocytosis.

**Abstract:**

Osteosarcoma (OS) is a primary malignant tumor of bone. Chemotherapy is one of the crucial approaches to prevent its metastasis and improve prognosis. Despite continuous improvements in the clinical treatment of OS, tumor resistance and metastasis remain dominant clinical challenges. Macropinocytosis, a form of non-selective nutrient endocytosis, has received increasing attention as a novel target for cancer therapy, yet its role in OS cells remains obscure. Benzethonium chloride (BZN) is an FDA-approved antiseptic and bactericide with broad-spectrum anticancer effects. Here, we described that BZN suppressed the proliferation, migration, and invasion of OS cells in vitro and in vivo, but simultaneously promoted the massive accumulation of cytoplasmic vacuoles as well. Mechanistically, BZN repressed the ERK1/2 signaling pathway, and the ERK1/2 activator partially neutralized the inhibitory effect of BZN on OS cells. Subsequently, we demonstrated that vacuoles originated from macropinocytosis and indicated that OS cells might employ macropinocytosis as a compensatory survival mechanism in response to BZN. Remarkably, macropinocytosis inhibitors enhanced the anti-OS effect of BZN in vitro and in vivo. In conclusion, our results suggest that BZN may inhibit OS cells by repressing the ERK1/2 signaling pathway and propose a potential strategy to enhance the BZN-induced inhibitory effect by suppressing macropinocytosis.

## 1. Introduction

Osteosarcoma (OS) is the most common primary bone tumor with relatively high malignancy in children and adolescents, susceptible to local invasion and metastasis [1]. For patients diagnosed with lung metastases, the 5-year survival rate drops to 20% or even lower [2,3]. At present, the standard treatment strategies for OS include chemotherapy, preoperative chemotherapy, tumor resection, and postoperative chemotherapy, among which chemotherapy is the preferred choice in clinical practice. Although the first-line therapeutic drugs in OS treatment have increased the survival rate of patients with OS, they exhibit numerous inevitable drawbacks such as drug resistance, low therapeutic effect on patients with metastasis, and non-selected toxic side effects, which are more likely to cause treatment failure [4,5,6]. Therefore, there is an urgent need to discover new chemotherapeutic drugs and treatment strategies for OS patients. Drug repurposing refers to the process of discovering new targets and expanding the potential application of old clinical drugs through thorough research. It is a fast, safe, and low-cost strategy for the development of anti-cancer drugs [7]. Benzethonium chloride (BZN), an FDA-approved antiseptic disinfectant, has been shown to fight against multiple cancers [8,9,10,11], such as lung cancer [9], head and neck squamous cell carcinoma [10], and liver cancer [11]. However, the potential anti-OS effect of BZN has not yet been reported.

Macropinocytosis is a non-selective endocytic pathway that internalizes extracellular fluid and its contents into cells via macropinosomes [12,13]. As a nutrient uptake pathway, macropinocytosis supports the growth and survival of cancer cells under nutrient stress, which is one of the significant reasons for multidrug resistance in cancer cells [14,15]. For instance, cancer cells can ingest amino acids, nucleotides, sugars, and fatty acids through macropinocytosis, thereby developing resistance to chemotherapeutic drugs that block anabolism [16]. It is worth noting that inhibition of macropinocytosis has been proved to enhance the sensitivity of cancer cells to some chemotherapy agents [16,17], highlighting that macropinocytosis is likely to be a potential target in cancer therapy.

In the present study, we sought to determine whether BZN had inhibitory effects on OS cell growth and metastasis in vitro and in vivo, along with its possible underlying mechanisms. Notably, we found that BZN significantly inhibited OS cell growth partly by repressing the ERK1/2 signaling pathway. Meanwhile, OS cells might employ macropinocytosis as a survival mechanism to counteract the antitumor effect of BZN. More meaningfully, inhibition of macropinocytosis potentiated the anti-OS effect of BZN in vitro and in vivo.

## 2. Materials and Methods

### 2.1. Cell Lines and Cell Culture

All cells were preserved by the Clinical Laboratory and Diagnostics Laboratory of Chongqing Medical University and cultured in a 37 °C, 5% humidified CO_2_ incubator. Human OS cell lines (143B and MG63) were maintained in MEM supplemented with 10% fetal bovine serum (ExCell Bio, Shanghai, China). Human bladder cancer T24 cells were cultured in RPMI-1640 containing 10% fetal bovine serum. Human non-small cell lung cancer A549 cells and cervical cancer Hela cells were cultured in DMEM containing 10% fetal bovine serum. All media were supplemented with 1% penicillin and streptomycin (100 IU/mL; Hyclone, UT).

### 2.2. Chemicals and Reagents

BZN, EIPA (NHE1 inhibitor), EHT1864 (Rac1 inhibitor), rottlerin (PKC inhibitor), and pamoic acid disodium (PAM, ERK1/2 activator) were purchased from MCE, and SB239063 (p38 inhibitor) from Selleck. BZN (10 mM), EIPA (40 mM), EHT1864 (20 mM), rottlerin (20 mM), and PAM (30 mM) were all dissolved in DMSO (Sigma, CA, USA) and stored at −80 °C. All reagents were purchased from Sigma-Aldrich (St. Louis, MO, USA) unless otherwise stated. All primary antibodies were purchased from Cell Signaling Technology (Boston, MA, USA), except for antibodies against MMP-2, MMP-9, Snail, p-JNK1/2/3 (Thr183 + Tyr185) (Affinity Biosciences, Cincinnati, OH, USA), and β-actin (Zoonbio Biotechnology, Nanjing, China).

### 2.3. Crystal Violet Staining

OS cells were seeded into 24-well plates (4 × 10^4^ cells/well). When cell density reached about 30%, cells were treated with different drugs at predetermined concentrations. After 24, 48, and 72 h of drug treatment, the culture medium was discarded. Then, cells were rinsed with PBS, fixed with 4% paraformaldehyde for 30 min, and stained with crystal violet dye for 15 min. For quantitative analysis, 500 µL of 10% glacial acetic acid was added to each well, followed by shaking for 10 min, and then the absorbance value was detected at a wavelength of 570 nm.

### 2.4. MTT Assay

OS cells were seeded into 96-well plates (2500 cells/well) and treated with different drugs at predetermined concentrations when cell density reached about 30%. After drug treatment for 24, 48, and 72 h, 10 µL of MTT was added to each well and incubated at 37 °C for 4 h. After the medium was removed, 100 µL of DMSO was added, followed by shaking at room temperature for about 10 min in the dark, and then the absorbance value was detected at a wavelength of 492 nm. All experiments were performed in triplicate.

### 2.5. Cell Morphology and Cell Proliferation Assay

143B cells were seeded into 24-well plates (2 × 10^4^ cells/well) and then treated with BZN (4 or 7 µM) and DMSO after adhering. Phase-contrast images were obtained under a light microscope at different time points (0, 24, 48, and 72 h), followed by MTT assays. Briefly, 50 µL of MTT was added to the corresponding wells and incubated at 37 °C for 4 h. After the medium was removed, 500 µL of DMSO was added and mixed well. Next, 200 µL of the lysate was added to a 96-well plate, and then the absorbance value was detected at a wavelength of 492 nm. All experiments were performed in triplicate.

### 2.6. Colony Formation Assay

OS cells were seeded at densities of 800 and 1500 cells/plate into 6-well plates, respectively. Cells were treated with different concentrations of BZN and cultured continuously for 7 days. After being rinsed with PBS, cells were fixed with 4% paraformaldehyde for 30 min and then stained with crystal violet. The number of clones was counted by using ImageJ software.

### 2.7. Wound Healing Assay

OS cells were seeded into a 6-well plate. When cell density reached about 90%, cells were scratched at a constant speed along the diameter of the 6-well plate with a 10 µL pipette tip to produce a uniform linear wound. Next, cells were washed with PBS and treated with different drugs at predetermined concentrations. At the indicated time points, each wound was randomly photographed, and the wound healing rate was calculated as follows: (wound width at 0 h − wound width at different time points)/(wound width at 0 h) × 100%.

### 2.8. Transwell Invasion Assay

Transwell chambers were coated with 60µL of Matrigel (diluted 20-fold with DMEM) and placed in a 37 °C incubator for 1 h. Next, 2.5 × 10^4^ OS cells in 400 µL of serum-free medium were added to the upper chamber, while 500 µL of 10% FBS containing different concentrations of BZN was added to the lower chamber. After 24 h of incubation, the non-invading cells on the upside of the upper chamber were removed with a cotton swab. The invading cells on the outside of the upper chamber were fixed with 4% paraformaldehyde, stained with crystal violet, and then photographed under a light microscope.

### 2.9. Flow Cytometry Analysis

Cells were seeded into 6-well plates. When cell density reached about 30%, cells were treated with different concentrations of BZN or DMSO for 24 h. After being digested into single cells with 0.25% trypsin, cells were washed twice with PBS and centrifuged at 1000 rpm for 5 min. Then, cells were re-suspended in 500 µL PBS and stained with Annexin V-FITC/PI kit for flow cytometric apoptosis detection. For cell cycle detection, cells were re-suspended in 100 µL PBS and 900 µL pre-cooling 75% ethanol, stored overnight at 4 °C, and followed by PI staining for detection and analysis.

### 2.10. Transmission Electron Microscope (TEM) Analysis

143B cells were treated with BZN (6 µM) for 36 h and then centrifuged at 800 rpm for 5 min after trypsinization. The cell suspension was transferred into a 1.5 mL EP tube, and about 2 × 10^6^ cells were collected by centrifugation at 1200 rpm for 10 min. Next, cells were fixed with 2.5% glutaraldehyde at 4 °C for 24 h. Then, cells were washed with 0.1 M sodium bicarbonate, fixed with 4% osmium tetroxide, dehydrated with acetone, infiltrated with a mixture of acetone and resin, and stained with lead citrate/uranyl acetate. The ultrastructures of cells and vacuoles were observed under a transmission electron microscope (Hitachi 7500, Tokyo, Japan).

### 2.11. Lucifer Yellow Incorporation Assay

After cells on the 24-well slides were treated with drugs for the indicated time (48 h for 143B cells, 24 h for A549 cells), the culture medium was removed. Cells were rinsed three times with PBS and further incubated with 1.5 mg/mL Lucifer yellow for 1 h at 37 °C. Then, the slides were rinsed with PBS three times, fixed with 4% paraformaldehyde for 30 min, and mounted with neutral resin. The representative images were acquired by a laser confocal microscope (Leica TCS SP8, Wetzlar, Germany) with an excitation wavelength of 428 nm.

### 2.12. Western Blot

143B cells were treated with different concentrations of BZN (4, 5, 6, or 7 µM) and DMSO for 24 h, and then lysed with RIPA cell lysis buffer (Boster, Wuhan, China) supplemented with 1% protease and phosphate inhibitors (Roche, Basel, Switzerland). Protein samples were separated by 10% sodium dodecyl sulfate-PAGE gel and transferred onto PVDF membranes. After blocking with 5% BSA for 2 h, the membranes were incubated with the corresponding primary antibodies (1:1000 dilution) at 4 °C overnight, followed by incubation with horseradish peroxidase-conjugated secondary antibody (1:5000 dilution) at 37 °C for an hour. Proteins of interest were visualized with the ECL kit and photographed with a gel imaging system. The gray values of the bands were analyzed with the Image Lab or ImageJ software.

### 2.13. Real-Time PCR

Total RNA was isolated by using the TRIZOL Reagent (Invitrogen, Carlsbad, CA, USA) according to the manufacturer’s instructions. The cDNA was synthesized by reverse transcription. Then, real-time quantitative PCR was performed as described [18]. The primers for the gene of interest are listed in Table 1 and were previously reported [19]. All PCR experiments were performed in triplicate. The relative quantification of gene expression was calculated using the 2^−ΔΔCq^ method and normalized to internal control, GAPDH expression.

### 2.14. OS Tumor Xenograft Model

BALB/c nude mice (female, 28 days, 12 ± 2 g) were purchased from Beijing HFK Bioscience and raised under specific pathogen-free (SPF) conditions in the Animal Center of Chongqing Medical University. Animals were provided with food and water ad libitum and subjected to a 12 h dark/light cycle throughout the experiment. All animal assays in this study were approved by the Ethics Committee of the First Affiliated Hospital of Chongqing Medical University. After the mice were acclimated for about 2 weeks, 60 µL of 143B suspension (2 × 10^7^ cells/mL) was injected into the proximal tibia of the mice. When tumors reached about 5 mm in diameter, mice were randomized into groups. To determine the inhibitory effect of BZN alone, all mice were randomly divided into five groups and given different doses of BZN (5 mg/kg, 10 mg/kg, or 15 mg/kg) or PBS by gavage every other day. To evaluate the combined effects of BZN and the macropinocytosis inhibitor EIPA on tumor growth, mice were divided into four groups and treated with Vehicle (CMC Na in PBS), BZN (5 mg/kg), EIPA (15 mg/kg) or BZN + EIPA for 17 days, respectively. The tumor size and body weight were recorded every 2 days. The tumor volumes were calculated using the following equation: V = (length × width^2^)/2. At the end of the animal assays, tumors and lungs were collected, fixed in 4% paraformaldehyde, and subjected to immunohistochemistry and hematoxylin-eosin (H&E) staining.

### 2.15. Hematoxylin-Eosin (H&E) Staining and Immunohistochemistry

Animal tissues were isolated, fixed with 4% paraformaldehyde, further embedded in paraffin, and then sectioned at 5 µm thickness. Tumor and lung tissue sections were deparaffinized and stained with hematoxylin-eosin (H&E). For immunohistochemistry, samples were deparaffinized in xylene, rehydrated in a gradient of 100%, 95%, and 75% ethanol, followed by antigen retrieval with sodium citrate. Next, endogenous peroxidase was blocked, and the sections were incubated with an appropriate amount of primary antibody at 4 °C overnight, reaction enhancer at 37 °C for 20 min, and the horseradish peroxidase-conjugated secondary antibody for 30 min. The bound peroxidase was visualized in DAB solution for 1–10 min. Finally, after counterstaining with hematoxylin, the sections were observed and photographed under a light microscope.

### 2.16. Statistical Analysis

The results were statistically analyzed by using GraphPad Prism 5.0. All experiments were performed in triplicate, and the raw data were expressed as mean ± standard deviation (SD). One-way ANOVA was used for comparison between multiple groups, and Tukey’s test was performed for comparison between any two groups. *p* < 0.05 was considered statistically significant.

## 3. Results

### 3.1. BZN Suppresses the Proliferation and Induces G1 Phase Arrest in OS Cells

**Figure 1:**
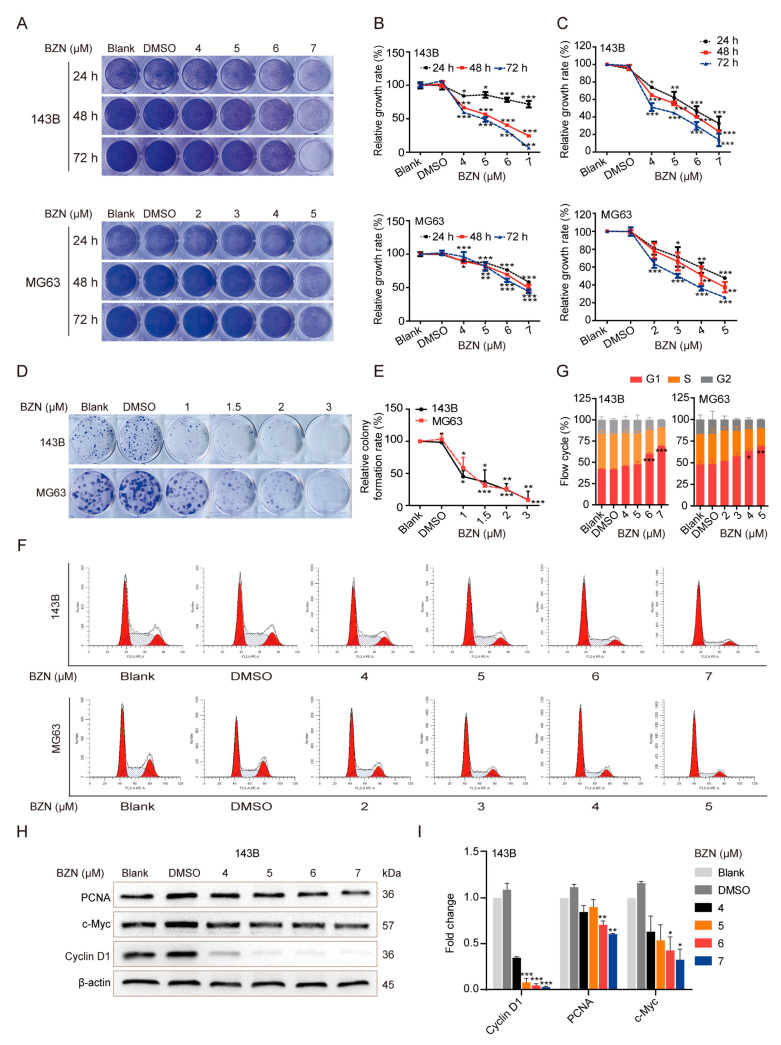
BZN suppresses the proliferation and induces G1 phase arrest in OS cells. (**A**) The effect of BZN on cell proliferation of OS cells (crystal violet staining). (**B**) Quantitative analysis of crystal violet staining. (**C**) The effect of BZN on cell viability of OS cells (MTT). (**D**) The effect of BZN on the colony formation ability of OS cells (colony formation assay). (**E**) Quantitative analysis of relative colony forming rate. (**F**,**G**) The effect of BZN on the cell cycle of OS cells (flow cytometry). (**H**) The effect of BZN on the protein levels of PCNA, c-Myc, and cyclin D1 in 143B cells. (**I**) Relative quantitative analysis of Western blot. Data are shown as mean ± SD from three independent experiments. * *p* < 0.05, ** *p* < 0.01, and *** *p* < 0.001 vs. DMSO group.

We first sought to investigate the effect of BZN on OS cell proliferation. Crystal violet staining showed that BZN significantly repressed the proliferation of OS cells (Figure 1A,B). The MTT assay further confirmed the inhibitory effect of BZN on OS cell viability (Figure 1C). In addition, colony formation assay validated that BZN visibly suppressed the in vitro colony formation ability of OS cells (Figure 1D,E). Next, we found that BZN reduced the protein levels of PCNA and c-Myc (Figure 1H,I), which are well-established proliferation markers. Subsequently, we sought to determine whether BZN interfered with the cell cycle of OS cells. By conducting flow cytometry analysis, we observed a prominent G1 phase arrest in BZN-treated OS cells (Figure 1F,G). Correspondingly, the protein level of cyclin D1, a pivotal regulator of the G1/S phase transition, was also decreased by BZN treatment (Figure 1H,I). These results indicate that BZN may suppress the proliferation and induce G1 phase arrest in OS cells.

### 3.2. BZN Inhibits OS Cell Migration and Invasion

**Figure 2:**
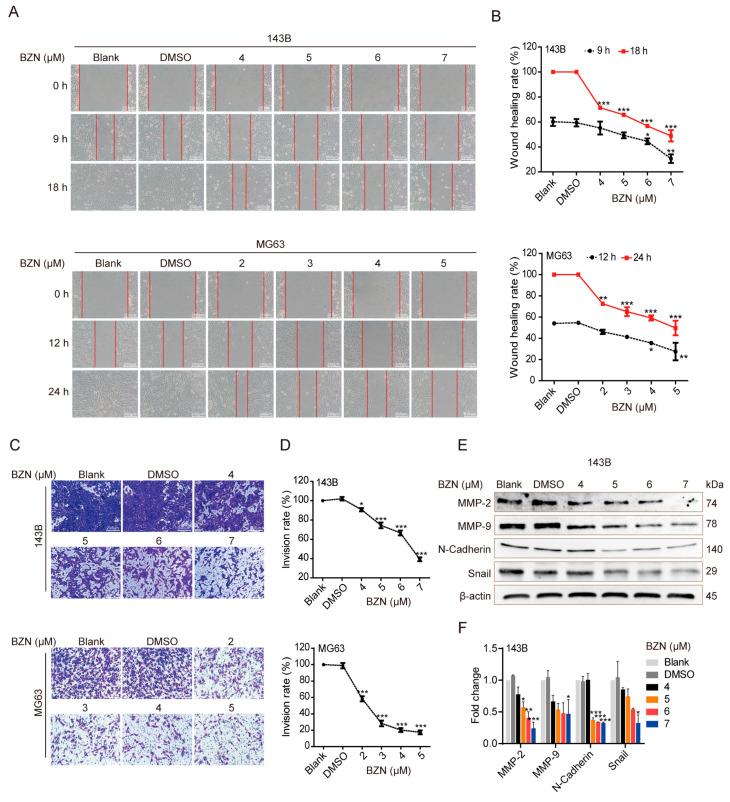
BZN inhibits the migration and invasion of OS cells. (**A**) The effect of BZN on the migration of OS cells (wound healing assay, scale bars: 200 µm). (**B**) Quantitative analysis of wound healing rate. (**C**) The effect of BZN on the invasion of OS cells (transwell assay, scale bars: 200 µm). (**D**) Quantitative analysis of invasion rate. (**E**) The effect of BZN on the protein levels of MMP-2, MMP-9, N-Cadherin, and Snail in 143B cells (Western blot). (**F**) Relative quantitative analysis of Western blot. Data are shown as mean ± SD from three independent experiments. * *p* < 0.05, ** *p* < 0.01, and *** *p* < 0.001 vs. DMSO group.

Next, we tested whether BZN affected the migration and invasion of OS cells. We found that the wound healing rate of BZN-treated OS cells was significantly reduced, which implies that BZN may suppress the migration of OS cells (Figure 2A,B). Using the transwell assay, we validated that the number of invasion cells penetrating across the matrigel-coated chamber was repressed significantly after BZN treatment (Figure 2C,D). These results suggest that BZN may repress OS cell migration and invasion. We then employed Western blot to determine whether BZN affected epithelial–mesenchymal transition (EMT), an essential process controlling cancer cell migration and invasion. We found that the protein levels of N-cadherin and Snail, two specific EMT markers, were significantly down-regulated by BZN. In addition, we validated that the protein levels of MMP-2 and MMP-9, the crucial executors of ECM degradation, were distinctly decreased in BZN-treated OS cells (Figure 2E,F). These results indicate that BZN may inhibit the migration and invasion of OS cells by suppressing EMT and MMPs.

### 3.3. BZN Had No Apparent Effect on the Apoptosis of OS Cells

**Figure 3:**
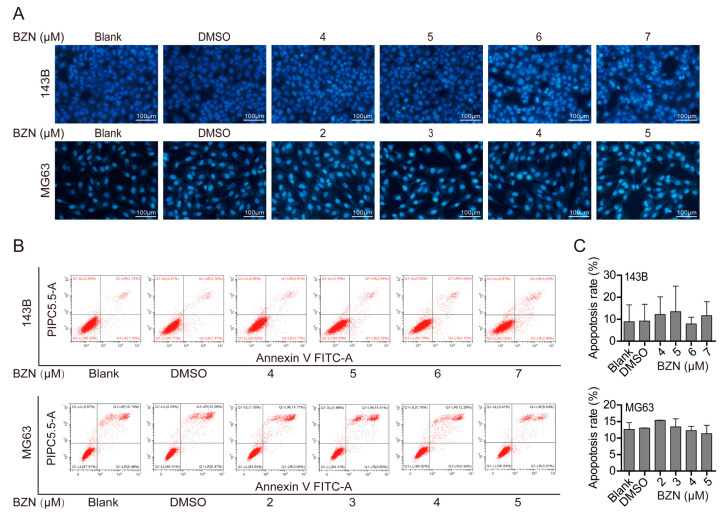
BZN-induced suppression of OS cell growth may not be related to apoptosis. (**A**) The effect of BZN on apoptosis of OS cells (Hoechst 33258 staining, scale bars: 100 µm). (**B**) The effect of BZN on apoptosis of OS cells (flow cytometry). (**C**) Quantitative analysis of apoptosis rates determined by flow cytometry. Data are shown as mean ± SD from three independent experiments.

Apoptosis is one of the most common ways through which chemotherapy drugs induce cancer cell death. We thus sought to clarify whether BZN induced apoptosis in OS cells. Surprisingly, BZN-treated OS cells exhibited neither typical morphological alterations of apoptosis in the nuclei (Figure 3A) nor increases in apoptosis rates (Figure 3B,C). These results suggest that BZN-induced OS cell death is non-apoptotic, although more studies are needed to further confirm this point.

### 3.4. BZN Exerts Anti-OS Effects via Repressing ERK1/2 Signaling Pathway

**Figure 4:**
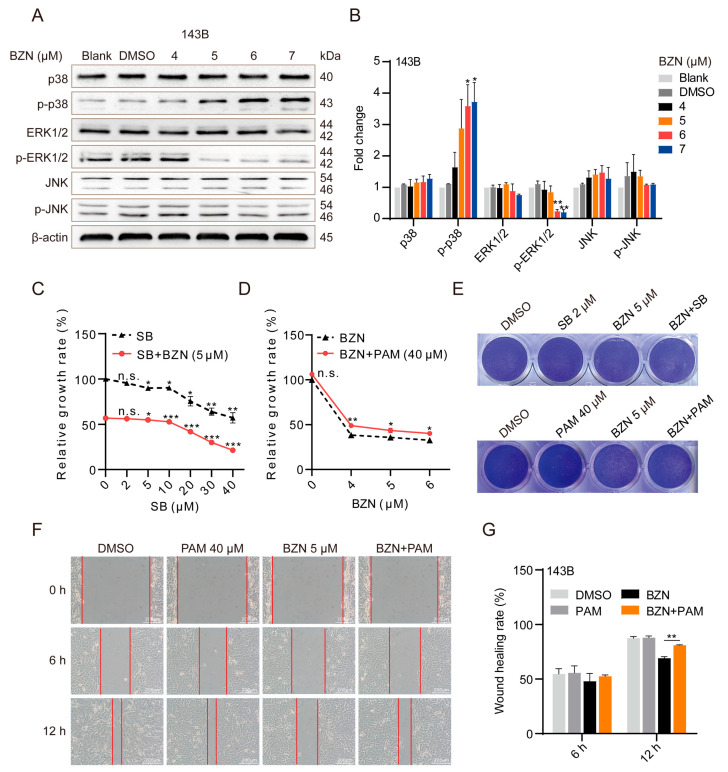
BZN-induced OS cell death involves the ERK1/2 signaling pathway. (**A**) The effect of BZN on p38, JNK, and ERK signaling pathways in 143B cells (Western blot). (**B**) Relative quantitative analysis of Western blot. (**C**) The effect of SB239063 in the presence or absence of BZN on the proliferation of 143B cells (MTT). * *p* < 0.05, ** *p* < 0.01, and *** *p* < 0.001 vs. SB = 0 µM (black line: DMSO group, red line: BZN group). n.s., not significant. (**D**) The effect of BZN in the presence or absence of PAM on the proliferation of 143B cells (MTT). * *p* < 0.05 and ** *p* < 0.01 vs. BZN group. n.s., not significant. (**E**) The effect of SB239063 or PAM in combination with BZN on the proliferation of 143B cells (crystal violet staining). (**F**) The effect of PAM combined with BZN on the migration of 143B cells (wound healing assay, scale bars: 200 µm). (**G**) Quantitative analysis of wound healing rate. Data are shown as mean ± SD from three independent experiments. ** *p* < 0.01 vs. BZN group.

Next, we sought to explore the mechanism underlying the BZN-induced inhibitory effect on OS cells. A previous study revealed that BZN suppressed the proliferation of lung cancer cells by activating the p38 signaling pathway [9]. Here, we found that BZN significantly promoted the phosphorylation of p38 (p-p38), indicating that the p38 signaling pathway was also activated by BZN in OS cells (Figure 4A,B). However, we found that the maximum tolerated concentration of SB239063 (p38 inhibitor) failed to prevent BZN-induced repression of OS cell proliferation (Figure 4C,E). Moreover, the high dose of SB239063 even significantly enhanced the BZN-induced suppression of proliferation (Figure 4C). The results suggest that the activation of the p38 signaling pathway may not mediate the inhibitory effect of BZN but may function as a negative regulator in BZN-induced repression of OS cells.

Considering that p38, ERK1/2, and JNK all belong to the mitogen-activated protein kinase (MAPK) signaling pathways, we also sought to analyze the changes in the ERK1/2 and JNK signaling pathways upon BZN treatment. We found that ERK1/2 phosphorylation (p-ERK) was significantly reduced after BZN treatment, while JNK phosphorylation (p-JNK) remained unchanged (Figure 4A,B). Moreover, after treatment of OS cells with PAM (ERK1/2 activator), BZN-induced cell death was considerably blocked (Figure 4D,E). In addition, PAM partially reversed BZN-induced migration suppression (Figure 4F,G). In summary, BZN may exert anti-OS effects at least partly by repressing the ERK1/2 signaling pathway.

### 3.5. BZN Inhibits Xenograft Tumor Growth and Metastasis of OS Cells In Vivo

**Figure 5:**
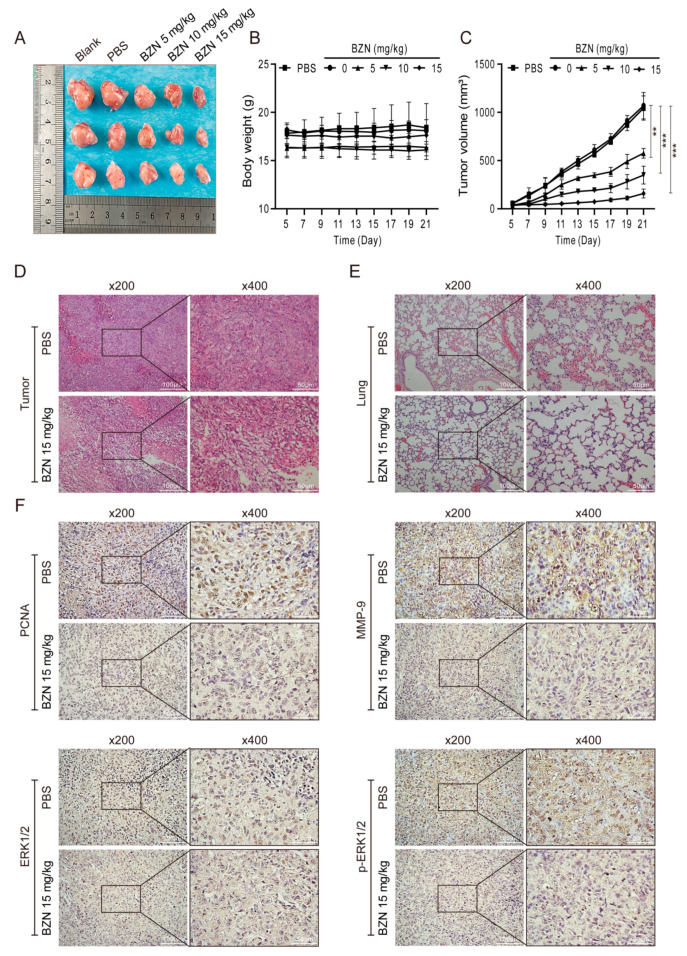
BZN inhibits tumor growth of 143B cells in vivo. (**A**) Representative OS samples retrieved from the xenograft tumor models. (**B**) The body weights of mice. (**C**) The average tumor volumes of the xenograft tumor models. (**D**) H&E staining of retrieved OS samples (×200, ×400). (**E**) H&E staining of retrieved lung tissues (×200, ×400). (**F**) The protein levels of PCNA, MMP-9, ERK1/2, and p-ERK1/2 of OS tissue sections (immunohistochemistry, ×200, ×400). Data are shown as mean ± SD from three independent experiments. ** *p* < 0.01 and *** *p* < 0.001 vs. PBS group.

Based on the in vitro results, we established a xenograft tumor model of OS cells to further verify the anti-OS effect of BZN in vivo. As exhibited in Figure 5A–C, the average tumor volumes in BZN-treated groups were significantly smaller than in the control group, while body weights were not altered perceptibly. Using H&E staining, we observed that BZN effectively reduced the malignant phenotypes (e.g., deeply stained nuclei and unbalanced nucleocytoplasmic ratio) of cells in xenograft OS samples, as well as micro-metastases in lung samples (Figure 5D,E). In addition, consistent with the in vitro results of Western blot, immunohistochemical staining showed that BZN decreased the protein levels of PCNA, MMP9, and p-ERK1/2 (Figure 5F). Taken together, these results suggest that BZN may suppress OS cell growth and metastasis in vivo.

### 3.6. BZN Induces Cytoplasmic Vacuolation in Tumor Cells

**Figure 6:**
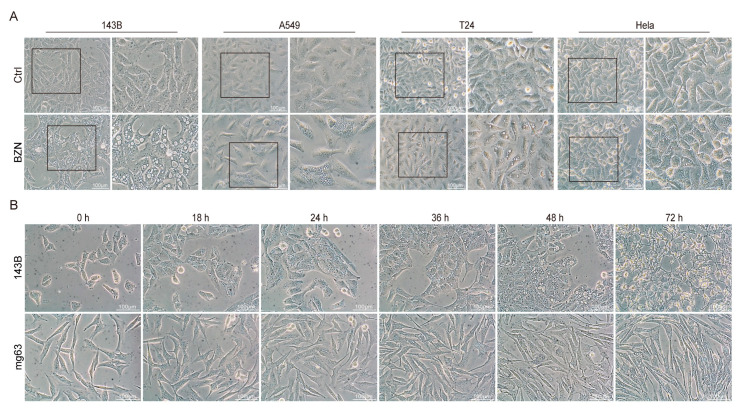
BZN induces cytoplasmic vacuolation in tumor cells. (**A**) The cell morphology of tumor cells (143B OS cells, A549 lung cancer cells, T24 bladder cancer cells, and Hela cervical cancer cells) treated with different concentrations of BZN (scale bars: 100 µm). (**B**) Phase contrast images of OS cells were obtained at the indicated time points after BZN (6 µM) treatment (scale bars: 100 µm).

We have proved that BZN may inhibit the growth and metastasis of OS cells in vitro and in vivo. It should be particularly emphasized that we could always observe a large number of phase-lucent vacuoles accumulated in the cytoplasm of BZN-treated OS cells. Moreover, we observed a similar phenomenon in other tumor cells upon BZN treatment, and the vacuoles in OS cells were the most significant (Figure 6A). These results indicate that the accumulation of vacuoles in BZN-treated tumor cells may be a general phenomenon, rather than an isolated event. After BZN treatment for about 18 h, a small number of vacuoles began to appear around the nuclei of OS cells. With the extension of time, the number of vacuoles increased and gradually merged into larger vacuoles. At the same time, the cell membranes fused, and the boundaries between cells were unclear. The size and number of vacuoles in 143B cells reached a peak at about 48 h, and then the vacuoles gradually condensed and became smaller until cells died. Compared with 143B cells, this morphological effect on MG63 cells changed more slowly and was less pronounced, while lasting longer, peaking at around 72 h (Figure 6B). These results suggest that BZN may induce the formation of cytoplasmic vacuoles in tumor cells.

### 3.7. BZN Induces Macropinocytosis in OS Cells

**Figure 7:**
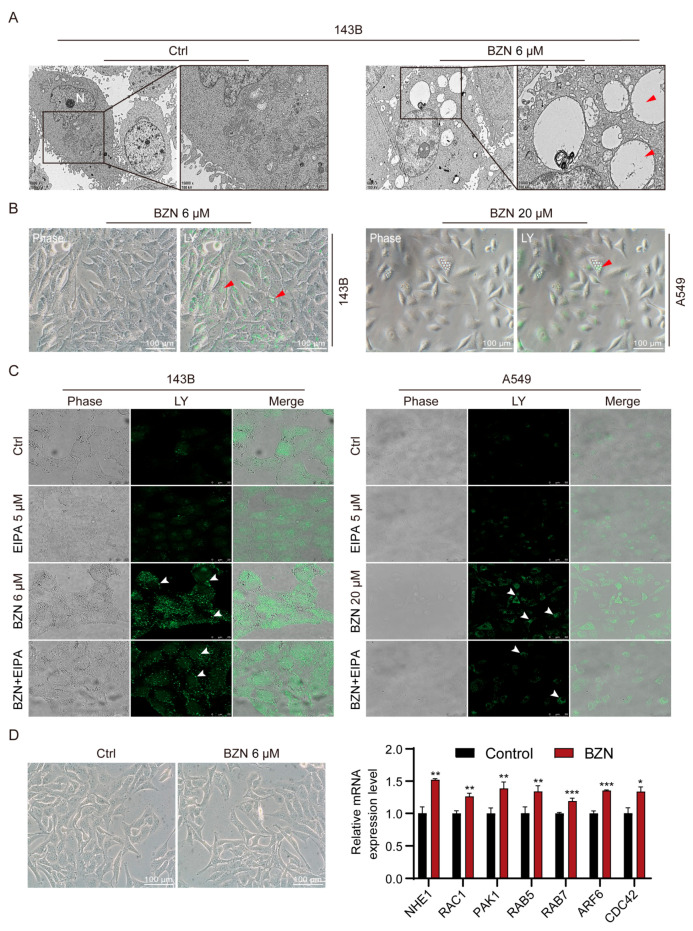
BZN induces macropinocytosis in OS cells. (**A**) Representative images of cellular ultrastructures in BZN-treated 143B cells (transmission electron microscopy, scale bars: 1/2 µm). Vacuolar ultrastructure is indicated by red arrows. N: Nucleus. (**B**) Representative images of Lucifer yellow (LY) in 143B OS cells and A549 lung cancer cells (scale bars: 100 µm). Red arrows indicate the LY localized in vacuoles. (**C**) The effect of EIPA on the LY uptake of 143B and A549 cells (laser confocal microscopy, scale bars: 25/50 µm). White arrows indicate the accumulated LY particles. (**D**) The mRNA expression levels of macropinocytosis-related genes (Q-PCR, right panel), and the corresponding cell morphology at this time (left panel, scale bars: 100 µm). Data are shown as mean ± SD from three independent experiments. * *p* < 0.05, ** *p* < 0.01, and *** *p* < 0.001 vs. Control group.

The vacuoles in BZN-treated OS cells especially attracted our attention. Next, we sought to determine the origin of these vacuoles. We performed transmission electron microscopy (TEM) analysis to visualize possible changes of cellular ultrastructures in BZN-treated 143B cells. As shown in Figure 7A, the cytoplasm of cells was filled with large-diameter vacuoles. These vacuoles with monolayer membranes had no inclusions and low electron density. There was neither agglutination/edge aggregation of chromatin in the nucleus nor significant swelling of organelles such as the endoplasmic reticulum and lysosomes. These morphological features were distinct from autophagy, apoptosis, and organelle swelling, but more similar to previously reported macropinocytosis [20,21]. Macropinocytosis is a form of endocytosis, albeit transient, typically characterized by the uptake of extracellular fluid phase. Lucifer yellow (LY), a commonly used fluid-phase tracer, is not permeable to cell membranes and can only be taken into cells through endocytosis [20]. As exhibited in Figure 7B, LY was accumulated in the vacuoles of BZN-treated 143B cells, which was similar to what was observed in lung cancer A549 cells, implying that the vacuoles in BZN-treated OS cells may be formed by endocytosis. 5-(N-ethyl-N-isopropyl) amiloride (EIPA) is a selective inhibitor of macropinocytosis and does not affect receptor-mediated endocytosis [22,23]. Noteworthily, EIPA significantly reduced the formation of vacuoles and the accumulation of LY in BZN-treated OS cells (Figure 7C). We surprisingly observed a similar phenomenon in BZN-treated A549 lung cancer cells as well (Figure 7C). These findings imply that BZN-induced vacuoles may originate from macropinocytosis, which may be universal in different types of cancer cells. Moreover, we found that the macropinocytosis-related genes were significantly up-regulated after BZN treatment (Figure 7D). Taken together, these results tentatively suggest that BZN may induce macropinocytosis in OS cells.

### 3.8. Inhibition of Macropinocytosis Enhances the Sensitivity of OS Cells to BZN In Vitro and In Vivo

**Figure 8:**
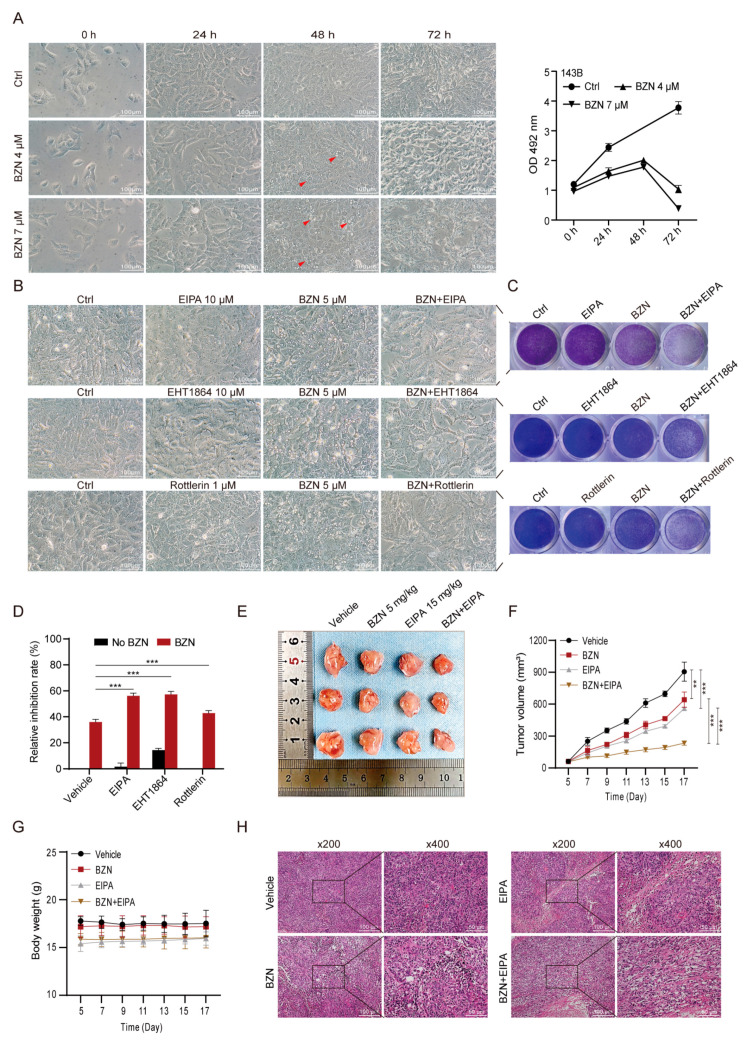
Macropinocytosis inhibitors enhance the anti-proliferative effect of BZN in vitro and in vivo. (**A**) Phase-contrast images of 143B cells treated with BZN (4 or 7 µM) at different time points (scale bars: 100 µm) and their corresponding cell proliferation (MTT). The red arrows refer to the cytosolic vacuoles. (**B**) The effect of EIPA, EHT1864, or rottlerin on BZN-induced macropinocytosis (scale bars: 100 µm). (**C**) The effect of EIPA, EHT1864, or rottlerin on the proliferation of 143B cells (crystal violet staining). (**D**) The effect of EIPA, EHT1864, or rottlerin on the proliferation of 143B cells (MTT). (**E**) Representative images of OS samples retrieved from the control group, BZN (5 mg/kg) group, EIPA (15 mg/kg) group, and combination group. (**F**) The tumor volume of the xenograft tumor. (**G**) The body weights of the xenograft mice. (**H**) H&E staining of retrieved OS samples (×200, ×400). Data are shown as mean ± SD from three independent experiments. ** *p* < 0.01 and *** *p* < 0.001.

To further explore the role of macropinocytosis in the growth and survival of BZN-treated OS cells, we tracked morphological changes and their corresponding proliferation in BZN-treated 143B cells at different time points. About 24 h after BZN treatment, the cytoplasmic vacuoles appeared in 143B cells. With the increase in vacuoles, 143B cells still maintained a relatively high proliferation rate from 24 to 48 h, and the cell density peaked around 48 h. From 48 to 72 h, the vacuoles gradually reduced, accompanied by a significant decrease in the cell proliferation rate (Figure 8A). The results show that macropinocytosis may be positively related to OS cell proliferation. Strikingly, we demonstrated that EIPA, EHT1864, and rottlerin, which are macropinocytosis inhibitors, significantly enhanced the anti-proliferative ability of BZN while mitigating BZN-induced macropinocytosis (Figure 8B–D). Taken together, these results suggest that inhibition of macropinocytosis may enhance the sensitivity of OS cells to BZN in vitro.

Next, we utilized xenograft tumor models of OS cells to confirm whether suppressing macropinocytosis also potentiated the anti-OS effect of BZN in vivo. We found that although low-dose BZN or EIPA alone exhibited a certain effect, the combination of these two drugs had a more significant inhibitory effect on the xenograft tumor growth of 143B cells (Figure 8E,F) without affecting the animal body weight appreciably (Figure 8G). We then performed H&E staining and demonstrated that the proliferation and nuclear/cytoplasmic ratio of OS cells in the BZN + EIPA combination group were dramatically lower than those in the single drug groups (Figure 8H). In conclusion, our findings suggest that inhibition of macropinocytosis may enhance the sensitivity of OS cells to BZN in vitro and in vivo.

## 4. Discussion

Over the past four decades, the treatment strategies for OS have long been neoadjuvant chemotherapy, adjuvant chemotherapy, and surgical resection [24]. The chemotherapy regimen for OS is mainly a combination of high-dose methotrexate, doxorubicin, and cisplatin (MAP) and etoposide and ifosfamide (EI) [25,26,27]. With the implementation of chemotherapy regimens, the survival rate in patients with the localized disease has improved, but for patients with recurrence or lung metastasis, tumor resistance and metastasis remain important causes of poor prognosis [6]. Considering that the traditional method of drug development is a time-consuming, costly and laborious process accompanied by a very low success rate, repurposing existing clinically used drugs is a more promising choice because the side effects and safety of selected agents have already been intensively tested [7]. In the area of anti-cancer drug discovery, drug repurposing in particular is receiving increasing attention. For example, All-trans retinoic acid, a drug commonly used in skin treatments, has been approved by the FDA for the differentiation therapy of acute promyelocytic leukemia [28]. Drug repurposing has also been used in the development of potential anti-OS agents. Ribavirin, an antiviral drug, has been reported to induce apoptosis of OS cells and increase chemosensitivity [29]. Our previous study also revealed that the anthelmintic drug nitazoxanide suppressed the proliferation of human OS cells [30]. In this current study, we demonstrated that the FDA-approved bactericidal disinfectant BZN suppressed the growth and metastasis of OS cells in vitro and in vivo. However, under the selective pressure imposed by BZN, OS cells may utilize macropinocytosis as a compensatory survival mechanism. Notably, inhibition of macropinocytosis with its inhibitors may enhance the anti-OS effect of BZN in vitro and in vivo.

The cell cycle is a tightly orchestrated process consisting of G1, S, G2 and M phases. All of these phases are precisely controlled by a series of regulatory components, among which cyclins are the most essential [31,32]. As a member of cyclins, cyclin D1 is a prominent regulator of the G1/S phase transition and contributes to increased proliferation. Actually, cyclin D1 is highly expressed and/or mutated in various cancers and is considered as a proto-oncogene [33,34]. Therefore, it is a better candidate for anti-cancer drug development, and agents that target cyclin D1 have shown promising potential in cancer treatment [35,36]. In this study, we found that BZN suppressed cell proliferation, decreased the protein level of cyclin D1, and induced G1 phase arrest of cell cycle in OS cells. As such, we propose that BZN may exert anti-OS properties by repressing cyclin D1, thereby inducing G1-phase arrest in OS cells.

EMT is the process during which cells lose their epithelial characteristics and acquire mesenchymal features. It endows cancer cells with formidable migration and invasion ability, thereby increasing the possibility of cancer cell metastasis. Snail, a pivotal EMT-transcription factor, can promote the expression of mesenchymal genes (e.g., N-cadherin), remodel the cytoskeleton and cell membrane, and thus induce EMT [37,38]. Noteworthily, EMT is often reversible, and drugs that can reverse the EMT process may have promising anti-metastasis abilities against cancer cells. ECM degradation is a fundamental step required for cancer growth and metastasis. The most vital executor of ECM degradation is the MMP family, whose expression is highly correlated with an increased metastasis potential. Among this family, gelatinases MMP-2 and MMP-9 can promote cancer metastasis by degrading collagen IV and gelatin, alone or in cooperation with other MMPs [39,40]. Here, we noticed that BZN treatment resulted in the suppression of migration and invasion in OS cells, along with the down-regulation of Snail, N-cadherin, MMP-2, and MMP-9. Accordingly, we proposed that BZN may inhibit the migration and invasion of OS cells by suppressing MMPs and reversing the EMT process. Anti-cancer drugs often kill cancer cells by triggering apoptosis. In lung cancer cells and hypopharyngeal squamous cancer cells, BZN significantly induced apoptosis [8,9]. Conversely, BZN protected triple-negative breast cancer cells from ceramide-induced apoptosis [41]. However, in the present study, we obtained no sufficient evidence suggestive of apoptosis in BZN-treated OS cells. We speculate that the inconsistent impact of BZN on apoptosis in these three types of tumor cells may be due to distinct genetic backgrounds.

MAPK signaling pathways are closely related to the occurrence and development of cancers, among which the ERK1/2, p38, and JNK signaling pathways are the most studied [42,43,44]. BZN has been shown to induce p38 phosphorylation, thereby promoting p38-mediated degradation of cyclin D1 to repress lung cancer cells [9]. Here, we found that although BZN promoted p38 phosphorylation, p38 inhibitor SB239063 failed to prevent cell death induced by BZN. Intriguingly, the anti-proliferative effect of BZN was significantly enhanced when the p38 inhibitor concentrations exceeded the maximum tolerated concentration. These results indicate that p38 activation may not be the mechanism directly mediating the anti-OS effect of BZN. Instead, it may be a spontaneous negative feedback adjustment to neutralize the anti-tumor effect of BZN on OS cells. Therefore, the combination of p38 inhibitor and BZN seems to have synergistic inhibitory effects against OS cells, which is worthy of further study. Conversely, unlike the unchanged ERK1/2 phosphorylation in lung cancer cells [9], BZN repressed the phosphorylation of ERK1/2, and PAM (ERK1/2 activator) partially reversed BZN-induced suppression of proliferation and migration in OS cells. Collectively, our findings suggest that BZN may exert anti-OS effects by repressing the ERK1/2 signaling pathway. Noteworthily, p38 may negatively regulate rather than mediate anti-tumor effect of BZN on OS cells. It should be emphasized that the roles of p38 and ERK1/2 in the BZN-induced anti-tumor effect are distinct between OS cells and lung cancer cells, although the exact mechanisms involved need to be further investigated.

Macropinocytosis represents a novel way of actin-dependent endocytosis by which cells ingest extracellular proteins, liquids, and particles, thus enhancing their adaptability to environmental fluctuations [20,45]. It plays multiple roles in various cellular physiological activities such as nutrient uptake, cell motility, angiogenesis, antigen presentation, and immune surveillance [46,47]. Although largely unexplored, the mechanism of macropinocytosis involves complex interaction networks consisting of a large number of signaling molecules such as Rac1 [45,48], PKC [49,50,51], and NHE1 [45,52,53,54]. Rac1, a small GTPase, can promote actin remodeling, which is an essential step of macropinocytosis, by interacting with PAK1 [45,48]. The importance of Rac1 to macropinocytosis has been demonstrated in several cell types. As proof, in human neuroblastoma SH-SY5Y cells, methamphetamine (METH)-induced macropinocytosis was highly related to the activation of Rac1 [55]. In addition to Rac1, PKC is also an important determinant of macropinocytosis. For instance, PKCδ regulated macropinocytosis by sequentially facilitating activation (dephosphorylation) of SSH1 and cofilin essential for actin rearrangement in macrophages [50]. The macropinocytosis process requires the activity of Na^+^/H^+^ exchange protein NHE1 to reach a critical pH near the plasma membrane, thereby promoting actin polymerization. Since NHE1 provides and maintains an appropriate submembrane alkaline pH for Rac1 activation, macropinocytosis is uniquely sensitive to Na^+^/H^+^ exchange inhibitors (such as amiloride and its derivative EIPA), which indirectly repress Rac1 by reducing submembrane pH [56]. In the OS model, previous studies have shown that OS cells (e.g., MG63, HOS, and U2OS) can produce macropinocytosis under some stimuli [21,57,58,59]. Constitutively active RAS triggered methuosis in U2OS cells through Rac1 activation and ARF6 inactivation [59], while the novel chalcone-like molecule (MIPP) involved the activation of RAB5 and RAB7 rather than Rac1 or ARF6 [21]. Nevertheless, the potential roles of macropinocytosis in OS are currently incompletely defined. In this study, we found that BZN induced macropinocytosis in OS cells. Additionally, we observed similar phenomena in other tumor cells, which indicates that BZN-induced macropinocytosis in tumor cells may be universal. Furthermore, we employed NHE1, Rac1, and PKC inhibitors commonly used in current research to initially search for possible signaling molecules and found that inhibition of NHE1, Rac1, and PKC mitigated BZN-induced macropinocytosis in OS cells. However, additional experiments (e.g., gene function studies and immunological detection) are needed to verify the roles of these signaling molecules and how their crosstalk affects macropinocytosis.

In recent years, macropinocytosis has received extensive attention as a new target for cancer therapy. However, it should be emphasized that macropinocytosis itself plays a dual role in cancers. Macropinocytosis is probably cancer-promoting because of its ability to provide additional nutrients for cancer cell growth and survival. Conversely, macropinocytosis may be cancer-suppressing as its hyper-activation may directly inhibit cancer cell growth through methuosis-dependent cell death [60]. At present, three macropinocytosis-based anti-cancer strategies have been proposed, including delivering chemotherapeutic drugs into cancer cells by macropinocytosis [61,62,63,64], hyper-activating macropinocytosis to induce methuosis in cancer cells [65,66,67,68,69], and inhibiting macropinocytosis to block the nutrient uptake of cancer cells [19,70,71,72,73,74]. By employing the last strategy, studies have shown that inhibition of macropinocytosis can enhance the anticancer effect of chemotherapy drugs commonly used in clinical practice. For example, in the human breast cancer model, the macropinocytosis inhibitor EIPA enhanced the anticancer effects of etoposide, cisplatin, tamoxifen, doxorubicin, and 5-FU [75,76]. Additionally, in human glioblastoma cells, inhibition of macropinocytosis led to a further increase in bevacizumab-induced cell death [17]. In this current study, we found that BZN-promoted macropinocytosis was beneficial to OS cells, which may partially counteract the inhibitory effect of BZN on OS cell growth. We thus speculated that OS cells may spontaneously produce macropinocytosis in response to BZN treatment, thereby neutralizing the anti-OS activity of BZN. Indeed, our speculation was strongly supported by a recent study. Sorafenib, a clinically therapeutic agent for hepatocellular carcinoma (HCC), could promote ferroptosis in HCC but simultaneously induce macropinocytosis leading to resistance. Adding macropinocytosis inhibitors to sorafenib improved the efficacy of sorafenib in HCC [77]. Accordingly, the antitumor effect of BZN was also enhanced by using the macropinocytosis inhibitors EIPA, EHT1864, and rottlerin. These findings indicate a potential strategy to achieve synergistic anti-OS effects through the combination of BZN and macropinocytosis inhibitors. In addition, although BZN is not an OS chemotherapy drug in clinical practice, we speculate that similar impacts may exist in the course of resistance to commonly used anti-OS drugs, and macropinocytosis may become one of the mechanisms of OS drug resistance. Thus, the synergistic anti-OS effects of macropinocytosis inhibitors in combination with more clinical chemotherapy drugs deserve to be further investigated.

## 5. Conclusions

In conclusion, we have demonstrated that BZN has anti-OS ability both in vitro and in vivo. Mechanistically, it may be related to the repression of the ERK1/2 signaling pathway. However, we found that BZN simultaneously induced macropinocytosis in OS cells. OS cells may utilize macropinocytosis to mitigate BZN-induced growth suppression and thus benefit their survival. The most meaningful finding is that inhibition of macropinocytosis significantly enhanced the sensitivity of OS cells to BZN. Overall, our study not only validates the inhibitory effect of the FDA-approved bactericidal disinfectant BZN against OS cells, but also provides a promising alternative strategy to enhance anti-OS activity by inhibition of macropinocytosis.

## Figures and Tables

**Table 1:** Primers used in quantitative real-time PCR.

Gene	Forward Primer (5′-3′)	Reverse Primer (5′-3′)
NHE1	TTCCCTTCCTTACTCGTGGTG	AATCGAGCGTTCTCGTGGT
RAC1	AAAACCGGTGAATCTGGGCT	AAGAACACATCTGTTTGCGGA
PAK1	GTCACAGGGGAGTTTACGGG	GCCTGCGGGTTTTTCTTCTG
RAB5	TACTTCTGGGAGAGTCCGCT	TTTGGGTTAGAAAAGCAGCCC
RAB7	GGTTCCAGTCTCTCGGTGTG	GAATGTGTTGGGGGCAGTCA
ARF6	CAACGTGGAGACGGTGACTT	TCCCAGTGTAGTAATGCCGC
CDC42	ACGACCGCTGAGTTATCCAC	TCTCAGGCACCCACTTTTCT

## Data Availability

The data used to support the findings of this study are available from the corresponding author upon reasonable request.
